# Practice of Medicine

**Published:** 1858-06

**Authors:** 


*Practice of Medicine.*—1. *Means of Detecting Albumen in Urine.* Dr. C. Gigon has made numerous experiments on the various tests of albumen in urine. He thinks he has ascertained the presence of this principle in normal urine, and he goes so far as to state that it is always to be found. This seems to be in opposition with Becquerel and Vernois, who think that albumen in urine is constantly a symptom of alteration of the kidneys. We have seen, long ago, that albumen taken in excess, as food, passes into the urine, and we could not admit the ideas of Messrs. Becquerel and Vernois. Now the facts put forward by M. Gigon show conclusively the error of these chemists. One of the causes of the discovery of M. Gigon consists in his having made use of chloroform as a test. It seems that chloroform is the most powerful agent of coagulation for the albumen; only *one thousandth* of albumen in water may be detected by it. This, M. G. says he has ascertained not only with urine, but with the serum of blood. It seems to me quite strange, if this be correct, that death does not take place more often when chloroform is inhaled. Mr. G. says that when there is but very little albumen in a solution, (urine or serum) after a few drops of chloroform have been added to the solution, it is unnecessary to shake it to obtain a precipitate. The following tests of albumen are disposed in a series beginning with the least sensitive: 1. Heat. 2. Pure alcohol. 3. Neutral acetate of lead. 4. Bi-cloride of mercury. 5. Nitrate of silver. 6. Nitric acid. 7. Sub-acetate of lead. 8. Tannin. 9. Creosote. 10. Chloroform. With the help of chloroform M. G. says he has ascertained that there is always in the urine of adults as well as of children a quantity of albumen, which he thinks to be the seven or eight hundredth part.

2. *Means of detecting Sugar in the Urine.* M. Leconte has published a paper, in which he tries to show that Dr. Blot has been mistaken in admitting that there is sugar, as a normal, or rather a constant element of urine in nursing women when they are in good health. Mr. Leconte attributes the decomposition of the salt of copper by the urine of these women to the uric acid. But Dr. Blot had ascertained the presence of sugar not only by the precipitate of oxide of copper in making use of Fehling's test, but also with the polarimeter, and by the result of fermentation. M. Leconte, however, is right in one thing, which is that uric acid may give a precipitate with all the tests containing a salt of copper. If we must attribute only to uric acid the large precipitate formed in treating the urine of nursing women with one of these tests, then the quantity of uric acid is very much increased in these women. A Dutch physician, Heynsius, has recently proposed a new view of this subject; he thinks that the substance mistaken for sugar and acting like it, in the urine, is a peculiar one, and only differing from sugar by some few characters. Whatever may be the interpretation of the positive facts discovered by Blot, their practical value remains entire. In a nursing woman in good health, the urine precipitates oxide of copper with Fehling's or Trommer's test, while if a serions disease exists, or is to come in a day or two, there is no more precipitate.—*New York Jour. Med.*

